# Supplementary material for: The novel reversible LSD1 inhibitor SP-2577 promotes anti-tumor immunity in SWItch/Sucrose-NonFermentable (SWI/SNF) complex mutated ovarian cancer
Source: PLoS One. 2020 Jul 10;15(7):e0235705. doi: 10.1371/journal.pone.0235705 (PMC7351179; doi:10.1371/journal.pone.0235705)
Supplement: S1 Raw images — Western blot analysis for SMARCA4 expression level in COV 434 pIND20 BRG1-2.7 after 1μM doxycycline daily treatment for 5 days (D = days). Total protein load was verified by LSD1 expression analysis. A) SMARCA4; B) LSD1. (PDF) [file pone.0235705.s001.pdf]

Supporting Information for:

The novel reversible LSD1 inhibitor SP-2577 promotes anti-tumor immunity in SWItch/Sucrose-NonFermentable (SWI/SNF) complex mutated ovarian cancer

Raffaella Soldi, Tithi Ghosh Halder, Alexis Weston, Trason Thode, Kevin Drenner, Rhonda Lewis, Mohan R. Kaadige, Shreyesi Srivastava, Sherin Daniel Ampanattu, Ryan Rodriguez del Villar, Jessica Lang, Hariprasad Vankayalapati, Bernard Weissman, Jeffrey M. Trent, William P.D. Hendricks and Sunil Sharma

Corresponding author: Sunil Sharma

Email: ssharma@tgen.org

This PDF file includes:

S1_raw_images. Original western blot and revert for Fig 6A. Western blot analysis for SMARCA4 expression level in COV 434 pIND20 BRG1-2.7 after 1μM doxycycline daily treatment for 5 days (D=days). Total protein load was verified by LSD1 expression analysis. A) SMARCA4; B) LSD1.

Original western blot and revert for Fig. 6A. Western blot analysis for SMARCA4 expression levels in COV 434 pIND20 BRG1-2.7 after 1 µM doxycycline daily treatment for 5 days (D=days). Total protein load was verified by LSD1 expression analysis. Western blot analysis was performed using an Odyssey Imager (Li-Cor Biosciences). A) SMARCA4; B) LSD1.

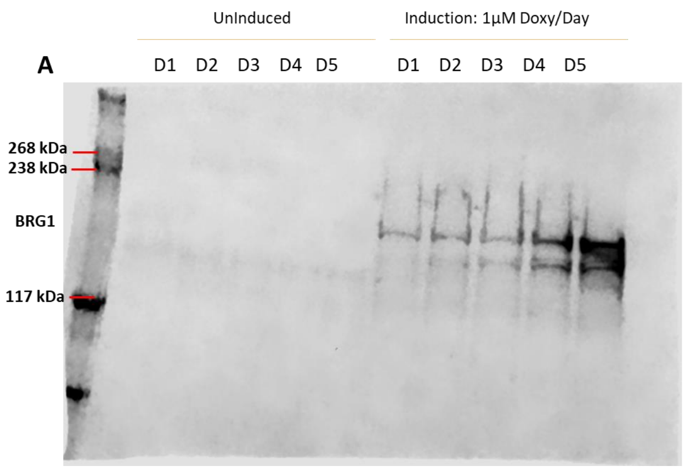

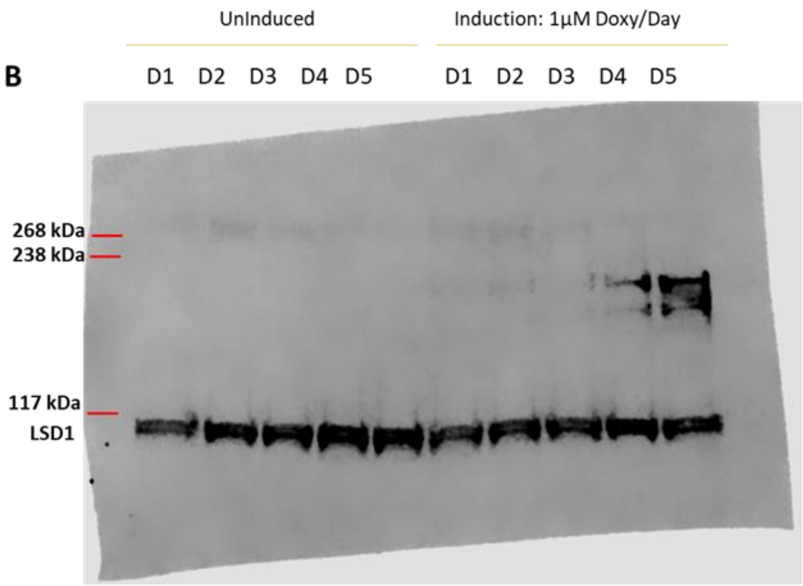
UnInduced
Induction: 1µM Doxy/Day
B
D1 D2 D3 D4 D5
D1 D2 D3 D4 D5
268 kDa
238 kDa
117 kDa
LSD1
