# Supplementary material for: The novel reversible LSD1 inhibitor SP-2577 promotes anti-tumor immunity in SWItch/Sucrose-NonFermentable (SWI/SNF) complex mutated ovarian cancer
Source: PLoS One. 2020 Jul 10;15(7):e0235705. doi: 10.1371/journal.pone.0235705 (PMC7351179; doi:10.1371/journal.pone.0235705)
Supplement: S1 Table — (PDF) [file pone.0235705.s002.pdf]

Supporting Information for:

# The novel reversible LSD1 inhibitor SP-2577 promotes anti-tumor immunity in SWItch/Sucrose-NonFermentable (SWI/SNF) complex mutated ovarian cancer

Raffaella Soldi, Tithi Ghosh Halder, Alexis Weston, Trason Thode, Kevin Drenner, Rhonda Lewis, Mohan R. Kaadige, Shreyesi Srivastava, Sherin Daniel Ampanattu, Ryan Rodriguez del Villar, Jessica Lang, Hariprasad Vankayalapati, Bernard Weissman, Jeffrey M. Trent, William P.D. Hendricks and Sunil Sharma

Corresponding author: Sunil Sharma

Email: ssharma@tgen.org

**This PDF file includes:**

**S2_Table_The cancer type acronym to full cancer type key from TCGA PanCancer Atlas**

S2:

| The cancer type acronym to full cancer type key from TCGA PanCancer Atlas | |
|---|---|
| **PanCancer Acronym** | **Cancer Type** |
| ACC | Adrenocortical Carcinoma |
| BLCA | Bladder Urothelial Carcinoma |
| BRCA | Breast Invasive Carcinoma |
| CESC | Cervical Squamous Cell Carcinoma |
| CHOL | Cholangiocarcinoma |
| COAD | Colorectal Adenocarcinoma |
| DLBC | Diffuse Large B-Cell Lymphoma |
| ESCA | Esophageal Adenocarcinoma |
| GBM | Glioblastoma Multiforme |
| HNSC | Head and Neck Squamous Cell Carcinoma |
| KICH | Kidney Chromophobe |
| KIRC | Kidney Renal Clear Cell Carcinoma |
| KIRP | Kidney Renal Papillary Cell Carcinoma |
| LAML | Acute Myeloid Leukemia |
| LGG | Brain Lower Grade Glioma |
| LIHC | Liver Hepatocellular Carcinoma |
| LUAD | Lung Adenocarcinoma |
| LUSC | Lung Squamous Cell Carcinoma |
| MESO | Mesothelioma |
| OV | Ovarian |
| PAAD | Pancreatic Adenocarcinoma |
| PCPG | Pheochromocytoma and Paraganglioma |
| PRAD | Prostate Adenocarcinoma |

| | |
|---|---|
| SARC | Sarcoma |
| SKCM | Skin Cutaneous Melanoma |
| STAD | Stomach Adenocarcinoma |
| TGCT | Testicular Germ Cell Tumors |
| THCA | Thyroid Carcinoma |
| THYM | Thymoma |
| UCEC | Uterine Corpus Endometrial Carcinoma |
| UCS | Uterine Carcinosarcoma |
| UVM | Uveal Melanoma |
