# Supplementary material for: The novel reversible LSD1 inhibitor SP-2577 promotes anti-tumor immunity in SWItch/Sucrose-NonFermentable (SWI/SNF) complex mutated ovarian cancer
Source: PLoS One. 2020 Jul 10;15(7):e0235705. doi: 10.1371/journal.pone.0235705 (PMC7351179; doi:10.1371/journal.pone.0235705)
Supplement: S2 Table — (PDF) [file pone.0235705.s003.pdf]

Supporting Information for:

The novel reversible LSD1 inhibitor SP-2577 promotes anti-tumor immunity in SWItch/Sucrose-NonFermentable (SWI/SNF) complex mutated ovarian cancer

Raffaella Soldi, Tithi Ghosh Halder, Alexis Weston, Trason Thode, Kevin Drenner, Rhonda Lewis, Mohan R. Kaadige, Shreyesi Srivastava, Sherin Daniel Ampanattu, Ryan Rodriguez del Villar, Jessica Lang, Hariprasad Vankayalapati, Bernard Weissman, Jeffrey M. Trent, William P.D. Hendricks and Sunil Sharma

Corresponding author: Sunil Sharma

Email: ssharma@tgen.org

This PDF file includes:

S3_Table_ List of primers used in qPCR

S3: List of primers used for qPCR

**S3**: qPCR primer sequences		
Target genes	Primer labels	Sequences
human HERV-K	HERVK-F	ATTGGCAACACCGTATTCTGCT
	HERVK-R	CAGTCAAAATATGGACGGATGGT
human ERVL	ERVL-F	ATATCCTGCCTGGATGGGGT
	ERVL-R	GAGCTTCTTAGTCCTCCTGTGT
human IFN-β	hIFNβ-F	GCCATCAGTCACTTAAACAGC
	hIFNβ-R	GAAACTGAAGATCTCCTAGCCT
human CXCL10	hCXCL10-F	TTCCTGCAAGCCAATTTTGTC
	hCXCL10-R	TCTTCTCACCCTTCTTTTTCATTGT
human ISG15	hISG15-F	CCTTCAGCTCTGACACC
	hISG15-R	CGAACTCATCTTTGCCAGTACA
human BRG1	hBRG1-F	CCTCTCTCAACGCTGTCCAACTG
	hBRG1-R	ATCTTGGCGAGGATGTGCTTGTCTT
human BRM	hBRM-F	GACGGCTCTCAACTCCAAAGCATAC
	hBRM-R	GACGGCGTTTCCTCTCCTGC
human PD-L1	hPD-L1-F	AAATGGAACCTGGCGAAAGC
	hPD-L1-R	GATGAGCCCCTCAGGCATTT
human GAPDH	hGAPDH-F	AACGGGAAGCTTGTCATCAA
	hGAPDH-R	TGGACTCCACGACGTACTCA
